# Anomalous Left Coronary Artery from Pulmonary Artery: An Important Cause of Ischemic Mitral Regurgitation in Children

**DOI:** 10.7759/cureus.4441

**Published:** 2019-04-12

**Authors:** Muhammad Kamran Younis Memon, Muneer Amanullah, Mehnaz Atiq

**Affiliations:** 1 Pediatrics and Child Health, Liaquat National Hospital and Medical College, Karachi, USA; 2 Pediatric Cardiac Surgery, National Institute of Cardiovascular Disease, Karachi, PAK; 3 Pediatric Cardiology, Liaquat National Hospital, Karachi, PAK

**Keywords:** mitral regurgitation, dilated cardiomyopathy, anomalous coronary artery from pulmonary artery (alcapa)

## Abstract

Introduction: Anomalous left coronary artery from the pulmonary artery (ALCAPA) is a rare congenital anomaly. The usual presentation in infancy is inconsolable crying or congestive cardiac failure, both due to myocardial ischemia. Survivors after infancy have improved left ventricular function but continue to have mitral regurgitation due to papillary muscle ischemia. The present study emphasizes the importance of unexplained mitral regurgitation as a clue to the diagnosis.

Patients and Methods: Patients with the diagnosis of ALCAPA operated between June 2017 and May 2018 were enrolled. Their ages at diagnosis, electrocardiography, and echocardiography findings were noted. A selective angiogram of the right coronary artery was done in all. Results of surgical reimplantation were analyzed. Postoperative data were collected, including ventricular function and mitral regurgitation.

Results: Six patients were included. Clinical signs of cardiac failure were present in two patients, and a systolic murmur was heard in all. The mean left ventricular ejection fraction was 52 ± 12%. Mitral regurgitation was present in all of the patients. The right coronary artery was dilated (Z score > 2.5) in all except one. Selective right coronary angiogram and cardiac computerized tomography angiogram (CTA) were performed in all. Coronary reimplantation was successfully done. Follow-up echocardiography showed improved left ventricular ejection fraction and degree of mitral regurgitation in all patients.

Conclusion: ALCAPA is an uncommon congenital anomaly, the diagnosis of which can be missed, particularly in late presenters. Unexplained mitral regurgitation should always raise the suspicion of this anomaly. Surgical intervention has excellent results with an improvement of left ventricular function and mitral regurgitation.

## Introduction

Anomalous left coronary artery from the pulmonary artery (ALCAPA) is a rare congenital anomaly occurring in 1:300,000 live births with a male preponderance, the ratio being 2:1 [1]. It is also called Bland-White-Garland syndrome, after clinicians who described the full spectrum of the disorder in 1933 and showed that the combined effects of the absence of normal coronary flow and a coronary steal produce profound myocardial ischemia leading to left ventricular dysfunction and mitral regurgitation [2]. Untreated, this anomaly carries a 90% mortality in the first year of life [3-4]. The usual presentation is in infancy with angina pain causing inconsolable crying or congestive cardiac failure, both due to myocardial ischemia. A subset may present after infancy with mitral regurgitation with or without other features of myocardial ischemia. Failure to diagnose and operate early leads to an overall grim outlook. We present our experience of an ALCAPA in infants and children presenting with mitral regurgitation as the predominant clinical and echocardiography feature, which improved postoperatively.

## Materials and methods

All consecutive patients with the diagnosis of an anomalous left coronary artery from pulmonary artery presenting from June 2017 to May 2018 were included. Clinical presentation was noted. The presence of congestive cardiac failure, inconsolable crying in infancy, and failure to thrive were noted. Clinical examination for poor growth, signs of congestive heart failure, and the presence of a murmur were sought. Electrocardiogram, echocardiogram, and selective right coronary angiogram were done in all patients. All patients had a computerized tomographic angiogram (CTA) done to evaluate the distance of the anomalous left coronary artery from the aortic root.

Surgical intervention included reimplantation of the left coronary artery to the aorta. Mitral annuloplasty was additionally performed in a 13-year-old with severe mitral regurgitation. All patients were discharged from the hospital.

## Results

Six patients with the diagnosis of an ALCAPA were included. None of the patients had a history of inconsolable crying episodes in early infancy. Clinical signs of cardiac failure were present in two patients, and an apical systolic murmur was heard in all patients. Preoperative and postoperative data are shown in Tables 1-2, respectively.

**Table 1:** Preoperative Patient Data IVS: interventricular septum; LAD: left anterior descending; LCA: left coronary artery; LV: left ventricle; MR: mitral regurgitation; PA: pulmonary artery; QTc: corrected QT interval; RCA: right coronary artery; RV: right ventricle; TR: tricuspid regurgitation

S #	Age at Diagnosis (yrs), Gender	Age at Surgery (yrs)	Echocardiography Findings	Preoperative ECG	Selective Coronary Angiography Findings
1	8 F	8	RCA z score +3, mild LV systolic dysfunction, mild to moderate MR, echogenic mitral valve papillary muscles, trace TR, color flow Doppler signals in RV wall, and IVS	Q waves in I, II	Dilated RCA, well-developed collateral circulation, retrograde filling the LCA, significant opacification of PA
2	15 M	15	RCA z score +3.5, moderate MR, low normal LV systolic function, color flow Doppler signals in RV wall, and IVS	Normal	Dilated RCA, well-developed collateral circulation, retrograde filling of LCA, opacification of PA. LAD artery arising from the left coronary sinus and connecting to PA like a fistula
3	9 M	10	RCA z score +5, normal LV function, moderate MR, trace TR, color flow Doppler signals in RV wall, and IVS	Normal	Dilated RCA, well-developed collateral circulation, retrograde filling the LCA, and significant opacification of PA
4	6 F	6	RCA z score +4, Normal LV systolic function and mild to moderate MR, trace TR, color flow Doppler signals in RV wall, and IVS	Q waves in I, II, V4, V5	Dilated RCA, well-developed collateral circulation, retrograde filling the LCA, and significant opacification of PA
5	13 F	13	RCA z score +5.5, low normal systolic LV function, severe MR, color flow Doppler signals in RV wall and IVS	LVH, no Q waves	Dilated RCA, well-developed collateral circulation, retrograde filling the LCA, significant opacification of PA
6	0.5 F	1.5	RCA z score +1, severe LV dysfunction and severe MR, moderate TR, color flow Doppler signals in RV wall and IVS	Prolonged QTc, no Q waves	Mildly dilated RCA, inadequately developed collateral circulation, retrograde filling the LCA, mild opacification of PA

**Table 2:** Operative and Postoperative Data ACC: aorta cross-clamp; CPB: cardiopulmonary bypass; LV: left ventricle; MR: mitral regurgitation; VT: ventricular tachycardia

S #	CPB time (min)	ACC time (min)	Ionotropic score	Postoperative arrhythmia	Duration of intubation (days)	Duration of hospital stay (days)	Postoperative echocardiography findings one-month postoperative follow-up
1	75	50	0	none	1	5	Normal LV systolic function, no MR, normal appearance of the reimplanted coronary artery
2	150	80	5	none	1	5	Normal LV systolic function, mild MR
3	80	40	0	none	1	6	Normal LV function, mild MR, reimplanted coronary artery mildly dilated, Z score +2.5
4	90	34	8	none	1	6	Normal LV systolic function, mild MR, normal appearance of the reimplanted coronary artery
5	135	115	0	none	1	6	Normal LV systolic function, moderate MR, normal appearance of the reimplanted coronary artery
6	130	95	5	One episode of VT	8	14	Severe LV systolic dysfunction (but improved), moderate MR, normal appearance of the reimplanted coronary artery

**Figure 1:**
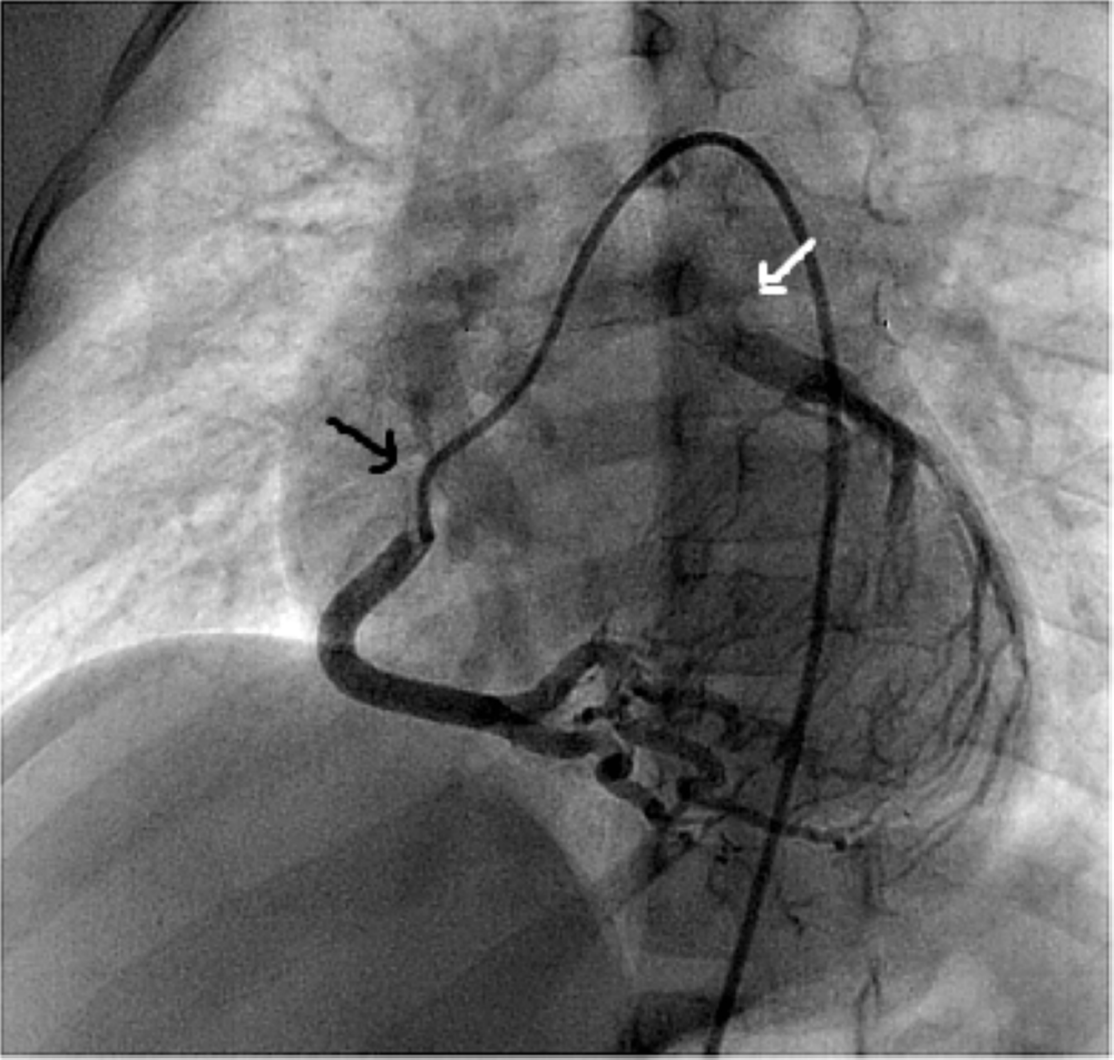
Selective right coronary angiogram (black arrow) in Patient #1, showing retrograde filling of the left coronary artery which opens into the pulmonary artery (white arrow)

Two patients had Q waves in the electrocardiogram, two had non-specific repolarization abnormalities, and in the remaining two, it was normal. The echocardiogram showed severe left ventricular dysfunction in only one patient and three had a low normal systolic function, whereas two had normal left ventricular function. The mean ejection fraction was 52 ± 12%. Mitral regurgitation of varying degrees was present in all patients. All patients also had color flow Doppler signals in the right ventricular wall and interventricular septum (IVS), indicating dilated collateral connections between the right and left coronary arteries. The right coronary artery was dilated on echocardiography in the short axis view (Z score >2.5) in all patients, except in Patient #6 who had poorly developed collaterals and severe left ventricular dysfunction. Selective right coronary angiogram was performed on all patients, and it demonstrated dilated right coronary artery with collaterals filling the left coronary system and retrograde opacification of the main pulmonary artery (Figure 1). CTA confirmed that the distance of the left coronary artery from the aortic root was close enough for reimplantation.

All patients underwent coronary reimplantation. The mean cardiopulmonary bypass time was 103 ± 22 minutes, and aorta cross-clamp time 71 ± 10 minutes. Mitral valve annuloplasty was performed in one child. Postoperatively mean inotropic score was 2.9 ± 0.3, and none had low cardiac output syndrome. All patients were discharged on beta blockers, angiotensin-converting enzyme inhibitors, diuretics, and aspirin. Follow-up echocardiogram performed one month later showed improved left ventricular ejection fraction and the degree of mitral regurgitation in all patients. 

## Discussion

ALCAPA causes insidious myocardial ischemia, leading to left ventricular systolic dysfunction. Three embryological theories have been put forth to explain this anomaly: the first proposes an abnormal disposition of the aortopulmonary septum; the second one suggests persistence of the left coronary buds on the pulmonary artery and involution of the aortic buds permitting anomalous development of that coronary from the wrong artery; the third one relates to a septation defect of the conotruncus tube [4-5]. The anomaly rarely involves the right coronary artery, left anterior descending artery, or both coronary arteries, the latter being incompatible with life [6]. Although reported to be fatal in infancy, children can survive past infancy into teenage and adulthood without symptoms. In them, an ALCAPA anomaly is an important cause of serious arrhythmias and sudden cardiac death [7].

The pathophysiology of the disorder relates to the pressure difference between the systemic and pulmonary arteries and the development of adequate collateral coronary circulation [8]. Four hemodynamic phases have been described. The first stage is in neonates with elevated pulmonary artery pressures supplying the left coronary artery with no myocardial ischemia. The second stage is when the pulmonary arterial pressure and vascular resistance fall and a reversal of flow from the left coronary artery occurs, resulting in a steal phenomenon. If collaterals from the right coronary artery are inadequate, hypoperfusion and myocardial ischemia develop. The third stage is the development of adequate collaterals connections between the two coronary circulations, resulting in better myocardial perfusion and improved myocardial function. The fourth stage is of normal pulmonary arterial pressure and vascular resistance with the left coronary circulation supplied by the right coronary artery and a left-to-right shunt ensues into the pulmonary artery. This causes subclinical myocardial ischemia leading to mitral valve regurgitation and sudden cardiac death due to arrhythmia [5-6]. The first two stages explain the pediatric type and the last two are found in the adult type of presentations. The echocardiographic clue to coronary artery collateral connections is the color flow Doppler signals in the right ventricular myocardium and interventricular septum.

Mitral regurgitation, as a disease entity, can be primarily due to a diseased mitral valve or its apparatus or secondary due to a diseased left ventricle [9]. The latter is due to pathological remodeling of the left ventricle due to either cardiomyopathy or coronary artery disease. Mitral regurgitation in the ALCAPA is due to 1) annular dilatation of the mitral valve secondary to ischemic left ventricular enlargement and dysfunction or 2) ischemic papillary muscle dysfunction, or both [10]. The former occurs in infancy and the latter in older children and adults. Understanding the mechanism is important for surgical management of the mitral valve.

Surgical intervention included coronary reimplantation in all of our cases. The patient with an accessory left anterior descending artery coronary connecting to the pulmonary artery as a fistula was ligated. Very few cases of additional coronary artery abnormalities have been reported previously [11]. After revascularization, the left ventricular function and size improved in five patients within one month. Mitral regurgitation improved in all patients, which may also have been due to improved perfusion of the mitral valve apparatus, as has also reported previously [12]. It is generally recommended not to address the mitral valve during the initial surgery as there is a high probability of mitral regurgitation improving with postoperative ventricular remodeling and reperfusion [10]. We performed an annuloplasty in the 13-year-old girl (Patient #5) due to severe mitral regurgitation.

## Conclusions

ALCAPA is an uncommon congenital anomaly. Diagnosis can be missed, particularly in late presenters, due to normalization of the left ventricular function. However, mitral regurgitation persists in this subset. Electrocardiographic abnormalities may not always be present in late presenters. Dilated cardiomyopathy with secondary mitral regurgitation is the common presentation in infancy. Surgical intervention has excellent results at all ages with improvement in left ventricular function and mitral regurgitation. The present study emphasizes the importance of unexplained mitral regurgitation as a clue to the diagnosis and its improvement after surgery. It is also imperative that a long-term follow-up of these patients is planned with an electrocardiogram and echocardiogram to look for arrhythmia, as well as left ventricular function and mitral regurgitation, respectively.
